# Trained quantity discrimination in the invasive red-eared slider and a comparison with the native stripe-necked turtle

**DOI:** 10.1007/s10071-024-01850-0

**Published:** 2024-03-26

**Authors:** Feng-Chun Lin, Pei-Jen Lee Shaner, Ming-Ying Hsieh, Martin J. Whiting, Si-Min Lin

**Affiliations:** 1 Department of Zoology, University of Otago, Dunedin, New Zealand; 2 School of Life Science, National Taiwan Normal University, No. 88, Tingzhou Road Section 4, Taipei, 116 Taiwan; 3 Department of Natural Resources and Environmental Studies, National Dong Hwa University, Hualien, Taiwan; 4 The Thinking Dog Vet Behaviour Team, Taipei, Taiwan; 5 School of Natural Sciences, Macquarie University, Sydney, NSW Australia

**Keywords:** Behavioral flexibility, Invasive species, Quantitative ability, Reptile, Weber’s law

## Abstract

Little is known about the behavioral and cognitive traits that best predict invasion success. Evidence is mounting that cognitive performance correlates with survival and fecundity, two pivotal factors for the successful establishment of invasive populations. We assessed the quantity discrimination ability of the globally invasive red-eared slider (*Trachemys scripta elegans*). We further compared it to that of the native stripe-necked turtle (*Mauremys sinensis*), which has been previously evaluated for its superior quantity discrimination ability. Specifically, our experimental designs aimed to quantify the learning ability as numerosity pairs increased in difficulty (termed fixed numerosity tests), and the immediate response when turtles were presented with varied challenges concurrently in the same tests (termed mixed numerosity tests). Our findings reaffirm the remarkable ability of freshwater turtles to discern numerical differences as close as 9 vs 10 (ratio = 0.9), which was comparable to the stripe-necked turtle’s performance. However, the red-eared slider exhibited a moderate decrease in performance in high ratio tests, indicating a potentially enhanced cognitive capacity to adapt to novel challenges. Our experimental design is repeatable and is adaptable to a range of freshwater turtles. These findings emphasize the potential importance of cognitive research to the underlying mechanisms of successful species invasions.

**Supplementary Information:**

The online version contains supplementary material available at 10.1007/s10071-024-01850-0.

## Introduction

Over the past two decades, reptiles have gained increased public attention, largely driven by the surge in reptile trade within pet markets (Marshall et al. 2020). This heightened visibility has, in recent years, been coupled with a growing concern for their welfare in captivity (Warwick et al. 2013, 2023). This shift in perception has been influenced by expanding knowledge about reptilian cognition (Wilkinson and Huber 2012) and learning capacities (Burghardt 1977; Szabo et al. 2021a). Advances in reptilian cognitive research benefit not just captive animals, but also inform our understanding of wild reptiles. Recent studies have integrated cognitive findings with the framework of ecology and evolution. For instance, investigations into the cognitive capabilities of urban-dwelling reptiles (Kang et al. 2018; Batabyal and Thaker 2019), invasive species (Damas-Moreira et al. 2020), and socially-oriented reptiles (Szabo et al. 2021b) offer insights. Findings that link cognitive aptitudes with specific ecological traits underscore the need to further dissect the relationship between reptilian cognition and ecology. This necessitates a deeper exploration of the cognitive abilities across diverse reptilian species.

More cognitive studies are not only beneficial to the welfare of captive reptiles, but also help us understand the biology of reptiles in the wild. Reptilian cognition has been studied within the context of ecology and evolution, considering traits like urban habitation (Kang et al. 2018; Batabyal and Thaker 2019), invasiveness (Damas-Moreira et al. 2020; Szabo et al. 2020), and social structure (Szabo et al. 2021b). Studies on reptilian cognition reveal correlations between cognitive abilities and ecological traits, underscoring the need to further understand this interplay across different species.

In nature, quantitative discrimination is pivotal to various animal behaviors such as foraging decisions, mate assessment, evaluating opponent numbers in confrontations, gauging brood parasitism risk, and assessing predation threats. Recognizing its significance in behavioral ecology and decision-making, recent studies have honed in on the quantitative discrimination capacities of reptiles. For example, wall lizards (*Podarcis sicula*) can differentiate between quantities of 2 and 4 (Miletto Petrazzini et al. 2018), gidgee skinks (*Egernia stokesii*) between 3 and 4 (Szabo et al. 2021b), Hermann’s tortoises (*Testudo hermanni*) between 3 and 4 (Gazzola et al. 2018), and the stripe-necked turtle (*Mauremys sinensis*) between 9 and 10 (Lin et al. 2021). The red-eared slider (*Trachemys scripta elegans*) has demonstrated an understanding of “relative quantity”, discerning stimuli of two different colors (Sun et al. 2023). Furthermore, a recent study also demonstrated relative quantity discrimination ability in African spurred tortoises (*Centrochelys sulcata*) (Tomonaga et al. 2023).

In our study, we delve deeper into the quantitative discrimination capabilities of the red-eared slider, listed among the top 100 most invasive species worldwide (Lowe et al. 2000; Stanford et al. 2020). The spread of this species has been detrimental to native turtle populations. In Italy, for instance, the native European pond turtle (*Emys orbicularis*) has seen population declines after sharing habitats with the red-eared slider (Cadi and Joly 2004). In Japan, female red-eared sliders, larger in size than their native counterparts, exhibit higher fecundity (Taniguchi et al. 2017). In some regions, rising climate change effects are anticipated to intensify habitat overlap conflicts between native and invasive turtles (Cerasoli et al. 2019). While there’s ample evidence showing the red-eared slider’s superiority over native species in certain ecological traits, such as foraging and basking (Cadi and Joly 2003; Polo-Cavia et al. 2008, 2011), it remains to be determined if this invasive species also surpasses native turtles in terms of cognitive abilities.

**Fig. 1:**
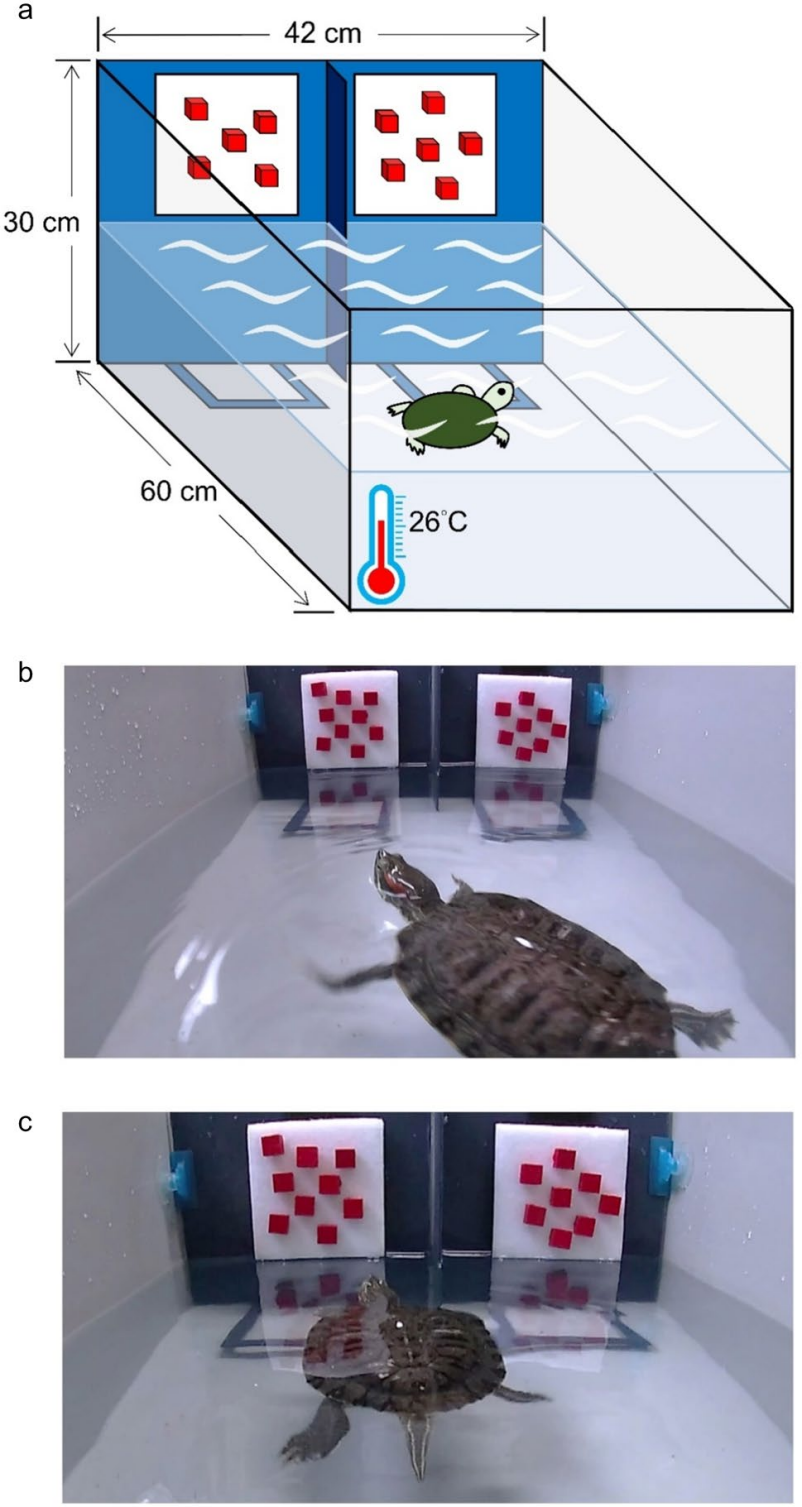
The experimental arena and quantitative stimuli. **a** The experiment arena was an acrylic tank (60 × 42 × 30 cm) filled with water to 15 cm depth. We mounted a GoPro (SPTM1) to the back wall of the tank and set a JVC camcorder (GZ-E10BU) on a tripod next to the tank. **b** We used wooden cubes (1.5 × 1.5 × 1 cm) colored with red acrylic paints (Mona, SG-203) on a white Velcro board (11 × 11 cm) as the quantitative stimuli. **c** Each turtle was trained to swim toward the stimuli and was rewarded with a food pellet when it reached the designated area (the square marked with blue stripes) for the correct (larger) quantity

In Taiwan, the red-eared slider, as an invasive species, threatens the survival of the native stripe-necked turtle (*Mauremys sinensis*) (Chen et al. 2000; Lee et al. 2019). In a prior study, we assessed the quantity discrimination ability of the stripe-necked turtle using a training method (Lin et al. 2021). The advantage of this training method (Fig. 1) lies in its repeatability and a standardized protocol, suitable for all freshwater turtles. We specifically examined the “ratio effect” as articulated by Weber’s law: as the ratio between quantities increases, it becomes numerically/quantitatively more challenging for the subject animals (Moyer and Landauer 1967; Meck and Church 1983; Butterworth et al. 2018). Our experimental designs aimed to qantify learning ability as the numerosity pairs increased in difficulty (referred to as fixed numerosity tests in this study) and the immediate, synchronized response when turtles were presented with varied challenges in rapid succession during the same tests (termed mixed numerosity tests).

Using the red-eared slider as our focal species and comparing it with the stripe-necked turtle, our objectives were: (1) to determine the quantity discrimination capability of the red-eared slider, and (2) to identify any differences in this ability between the invasive slider and the native turtle that might account for the dominance and survival of the former. Additional insights into the red-eared slider’s cognition not only augment our basic understanding of reptilian cognitive processes but also elucidate the relationships among cognition, ecology, and the underlying mechanisms of invasion biology.

## Materials and methods

### Subjects

We adopted the same methodology as described in Lin et al. (2021). We housed 12 juvenile red-eared sliders (approximately 2 years old, sourced from certified breeders) in a semi-natural yard spanning 60 square meters. For training and experimentation, the turtles were relocated to an indoor area (200 × 160 cm) equipped with a rectangular pool (120 × 80 × 30 cm). The surrounding temperature was maintained at 26 °C, and natural lighting was used. Turtles were fed on alternate days using commercial food pellets, which also served as rewards during the experiments. All the procedures used in this study followed protocols approved by Institutional Animal Care and Use Committee (IACUC) of National Taiwan Normal University (license No. 107029).

### Pre-training

The objective of the pre-training was to condition the turtles to associate the side displaying a greater number of red cubes with a reward (i.e., food pellets). This conditioning comprised four critical stages:

1. Association of Food with Tweezers: The primary aim was to alleviate the turtles’ apprehension towards the experimenter. Using classical conditioning, turtles were trained to associate food with tweezers, encouraging them to actively approach the tweezers.
2. Stimulus-Reward Association: Here, food was linked to a white board decorated with three red cubes, positioned behind the tweezers. This setup prompted the turtles to swim towards the board to obtain the food rewards.
3. Discriminating Higher Quantity: At this stage, turtles were presented with two side-by-side boards: one displaying a single red cube, and the other three red cubes. The experimenter randomly alternated the boards’ positions. Turtles received rewards only when they directed themselves towards the board showcasing three cubes.
4. Choice in a Designated Zone: Turtles were trained to navigate to a predetermined location within the tank before making their selection (Fig. 1). They were expected to repeat this behavior to earn subsequent rewards.

Throughout these training stages, correct responses were positively reinforced with food rewards and no punishment was used.

The time taken for a turtle to complete these four steps varied among individuals, typically requiring a total of about 60 days of continuous training (15 trials per day). For the red-eared sliders, we began relocating the turtles to the experimental area in mid-June 2020, with experiments commencing in mid-August 2020.

Out of the initial 12 turtles, five did not meet all the criteria from the four steps and were therefore excluded from subsequent training. Of the remaining seven turtles, one did not pass the initial tests in the subsequent experiments. Consequently, a total of six sliders successfully completed both the pre-training requirements and proceeded to participate in Experiment 1 and Experiment 2. This attrition rate mirrors our previous study with the stripe-necked turtle (Lin et al. 2021).

### Experiment 1: fixed numerosity tests

Experiment 1 (Video S1) consisted of a series of fixed numerosity tests in which the difficulty of the tests increased over time. In this setup, the turtles faced only one pair of numerosities per day. They underwent 20 trials daily, with the cube layout and the correct side randomized for each trial. After 5 consecutive days, totaling 100 trials for a given numerosity pair, the experiment transitioned to the next numerosity pair on the 6th day. According to our design, the difficulty of the numerosity pairs escalated based on Weber’s law (Butterworth et al. 2018). The sequence began with 1 vs 3 (ratio = 0.33, from Aug. 17th to 21st, 2020), progressing to 2 vs 4 (ratio = 0.50, Aug. 22nd to 26th), then 3 vs 4 (0.75, Aug. 27th to 31st), followed by 4 vs 5 (0.80, Sep. 1st to 5th), and culminating in 6 vs 7 (0.86, Sep. 6th to 10th). All these procedures mirrored those employed for the stripe-necked turtles in our prior study (Lin et al. 2021).

### Experiment 2: mixed numerosity tests

During the mixed numerosity tests (Videos S2 and S3), each turtle was presented with a set of 10 to 11 distinct numerosity pairs daily, reflecting a variety of challenges in rapid succession. This contrasts with the fixed numerosity tests. Furthermore, each of these pairs was presented twice in a single day, leading to a total of 20 to 22 trials. These trials were conducted over 5 consecutive days (forming a single phase). While the set of 20 to 22 trials remained consistent in a phase, the order in which the numerosity pairs were presented was randomized across the 5 days. In Phase 1, running from Sep. 27th to Oct. 1st 2020, the numerosity pairs combined smaller numbers (ranging from 1 to 5) and had ratios of difference between 0.2 and 0.8 (as detailed in Table S1). During Phase 2, from Oct. 2nd to 6th, the pairs incorporated at least one larger number (between 6 to 10), introducing more challenging pairings such as 6 vs 8, 6 vs 9, and 8 vs 10. In Phase 3, from Oct. 7th to 11th, the ratio range extended to 0.9, with pairs like 7 vs 9, 8 vs 9, and 9 vs 10 (Table S1). With this design, a turtle experienced 10 trials for each numerosity pair over the duration of the 5-day phase. The arrangement of these pairs, from low to high ratios, provided an opportunity to assess the turtles’ performance across different levels of difficulty.

### Statistics in fixed numerosity tests

To determine if the turtles’ quantitative performance in the fixed numerosity tests (Experiment 1) surpassed random chance, we computed the success rate for each turtle and for each numerosity pair. Here, the success rate was defined as the proportion of successful trials out of the total trials conducted over 5 continuous days. Binomial tests were applied at the individual turtle level (with the null hypothesis postulating a success rate greater than 50% of the total number of trials). For the group as a whole, we utilized the Wilcoxon signed rank tests (with the null hypothesis suggesting a median success rate exceeding 0.5). In a bid to understand the turtles’ performance in relation to the varying degrees of difficulty (as denoted by the ratio), we proceeded to fit a linear mixed model. This model integrated the overall success rate of the five designated pairs (1 vs 3, 2 vs 4, 3 vs 4, 4 vs 5, and 6 vs 7) and their interactions as fixed effects. The Subject, or turtle ID, was incorporated as a random effect.

### Statistics in mixed numerosity tests

According to Lin et al. 2021, in the mixed numerosity tests (Experiment 2), when faced with more challenging tasks and increased complexity, individual differences among the turtles become more pronounced. Therefore, we employed linear models to analyze the overall success rate, including Subject (turtle identity), Ratio, and their interactions as fixed effects. Recognizing that the turtles’ abilities could progressively improve throughout the phases, the Phase (1, 2, 3) was incorporated as a covariate in the models. We initiated our analysis with the full model (success rate = Subject + Ratio + Subject × Ratio + Phase) and refined it to identify the best-fit model for parameter estimates, guided by the likelihood ratio test (LRT).

### Comparison between the two turtles

In Experiment 1, we evaluated the performance of the red-eared sliders in relation to that of the native stripe-necked turtles (refer to Table S2) previously detailed in Lin et al. (2021). A linear mixed model was formulated for the total success rate of each pair, integrating Species (turtle species), Ratio of the five pairs (1 vs 3, 2 vs 4, 3 vs 4, 4 vs 5, and 6 vs 7), and their interactions as fixed effects, and with Subject (turtle ID) as a random effect.

For Experiment 2, we constructed a linear mixed model for the overall success rate of each pair across the three phases. This encompassed Species (turtle species), Ratio of all test pairs, and their interactions as fixed effects, with Subject (turtle ID) and Phase being treated as random effects. To delve into the effect of ratio across varying phases, the three phases were individually assessed with distinct models for the total success rate. Each of these models included Species (the two turtle species), Ratio of all test pairs, and their interaction as fixed effects, accompanied by Subject (turtle ID) as a random effect.

All statistical analyses were conducted in R v3.6.1.

### Ethical note

All the procedures used in this study followed protocols approved by Institutional Animal Care and Use Committee (IACUC) of National Taiwan Normal University (license No. 107029).

## Results

### Performance of the sliders

After the training process, six red-eared sliders successfully chose the higher quantity from the two stimuli in the fixed numerosity tests. In Experiment 1 (fixed numerosity tests), the turtles’ performance significantly surpassed random chance both at the individual level (binomial tests; P < 0.001 in the majority of cases, as shown in Table 1) and at the group level (Wilcoxon signed rank tests; P < 0.01 for all 5 numerosity pairs, as shown in Table 1). All six individuals navigated the most challenging pairing (6 vs 7) with a median success rate of 0.677. This indicates they successfully internalized the “greater than” concept, which they subsequently employed to discern unfamiliar quantities in the mixed numerosity tests.

**Table 1:** Performance (correct/total trials) of the red-eared sliders in individual and group levels

Pair	Ratio	Individual level (binominal tests)						Group level (Wilcoxon tests)
		TS11	TS12	TS13	TS16	TS21	TS22	Median success rate
Experiment 1 (fixed numerosity tests)								
1 vs 3	0.33	69/100***	87/100***	64/101**	76/100***	69/100***	81/100***	0.725**
2 vs 4	0.50	66/100***	79/099***	76/100***	75/100***	70/101***	83/101***	0.755**
3 vs 4	0.75	66/100***	77/100***	69/100***	73/100***	68/100***	75/100***	0.710**
4 vs 5	0.80	73/101***	76/100***	67/101***	68/100***	80/100***	82/100***	0.741**
6 vs 7	0.85	67/100***	69/101***	63/101**	62/100*	73/100***	82/100***	0.677**
Experiment 2 (mixed numerosity tests; only the highest two pairs were shown)								
8 vs 9	0.89	8/10	7/10	10/10***	8/10	8/10	9/10**	0.800**
9 vs 10	0.90	9/10**	7/10	5/10	6/10	9/10**	9/10**	0.800**

Each subject in each testing phase underwent 99–101 trials. The minor variance in trial numbers is due to occasional errors made during the manual operation process, such as administering one more or one less trial in a given day, and cannot be rectified afterward

*P < 0.05; **P < 0.01; ***P < 0.001

In Experiment 2 (mixed numerosity tests), the sliders were presented with notably challenging pairs, such as 8 vs 9 (r = 0.89) and 9 vs 10 (r = 0.90) (Table 1). Among the six turtles, two mastered the 8 vs 9 tests, with one individual (TS13) achieving a flawless 100% correct rate. Similarly, three turtles adeptly navigated the 9 vs 10 tests, registering a commendable 90% accuracy rate. When assessing the collective performance, the group demonstrated the capability to differentiate both the 8 vs 9 and 9 vs 10 pairings (Wilcoxon tests; P < 0.01 for both sets).

**Fig. 2:**
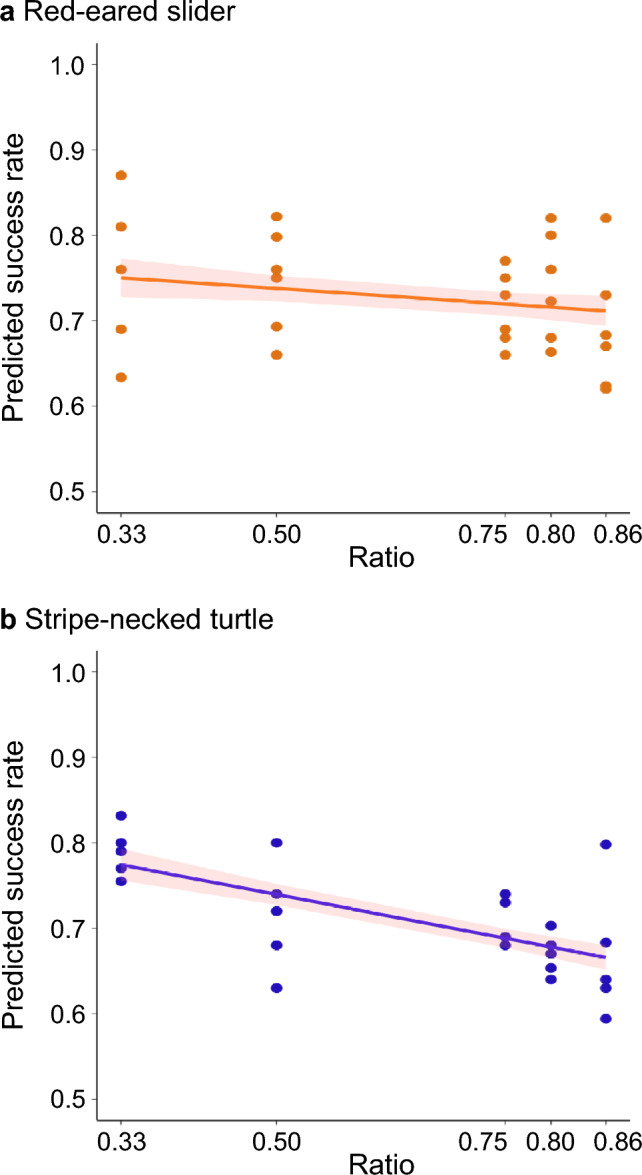
Comparison of ratio effects between red-eared slider (*Trachemys scripta elegans*, orange) and stripe-necked turtle (*Mauremys sinensis*, blue) in the fixed numerosity tests (Experiment 1). Each numerosity pair was tested 100 times on each individual, starting from the lowest ratio (1 vs 3, ratio = 0.33) to the highest ratio (6 vs 7, ratio = 0.86)

For the fixed-numerosity tests, the “Ratio” negatively influenced performance, though this trend didn't attain statistical significance (P = 0.11, as detailed in Table 2 and Fig. 2a). Meanwhile, in the mixed-numerosity tests, only Subject and Ratio persisted in the best-fit model. This suggests variations in performance across individuals and a decreasing success rate as the ratio increased (P < 0.001, Table 3). Other parameters like Phase and their corresponding interactions were omitted in the best-fit model.

**Table 2:** Ratio effects and cross species comparison in fixed numerosity tests

Ratio effect of red-eared slider					
Fixed effect	Estimate	SE	df	t	P
(Intercept)	0.77426	0.03561	22.75083	21.746	< 0.001***
Ratio	− 0.07296	0.04387	23	− 1.663	0.11

Random effect	variance	SD			
Subject	0.00229	0.04788			
(Residual)	0.00233	0.04824			

Comparison of ratio effects between the sliders and the turtles					
Fixed effect	Estimate	SE	df	t	P
(Intercept)	0.84302	0.03692	49.01202	22.835	< 0.001***
Species TS (vs MS)	− 0.06876	0.04986	48.94893	− 1.379	0.17419
Ratio	− 0.20726	0.04939	42	− 4.197	0.00014***
Species TS: ratio (vs MS: ratio)	0.1343	0.06666	42	2.018	0.05001

Random effect	variance	SD			
Subject	0.00125	0.03528			
(Residual)	0.00241	0.04905			

TS: the red-eared slider (*Trachemys scripta elegans*); MS: the stripe-necked turtle (*Mauremys sinensis*)

**Table 3:** Best-fit model (subject + ratio) of the red-eared slider in mixed numerosity tests

	Estimate	SE	T	p
(Intercept)	0.82947	0.03436	24.142	< 0.001***
Ratio	− 0.20555	0.04602	− 4.467	< 0.001***
TS11 (vs TS21)	0.04062	0.03365	1.207	0.22882
TS12 (vs TS21)	0.07813	0.03365	2.322	0.02133*
TS13 (vs TS21)	0.07812	0.03365	2.322	0.02133*
TS16 (vs TS21)	0.06875	0.03365	2.043	0.04244*
TS22 (vs TS21)	0.11562	0.03365	3.436	0.00073***

*P < 0.05; **P < 0.01; ***P < 0.001

### Comparison between the two species

In the fixed numerosity test, the stripe-necked turtle exhibited a significant ratio effect (P = 0.00038, Table S2; Fig. 2b), indicating diminished performance when faced with high-ratio (more challenging) pairings. Conversely, the sliders did not exhibit such significance (Fig. 2a). When comparing the ratio effects of the two, there was a significant difference between the species (t = 2.018, P = 0.05001, Table 2).

**Fig. 3:**
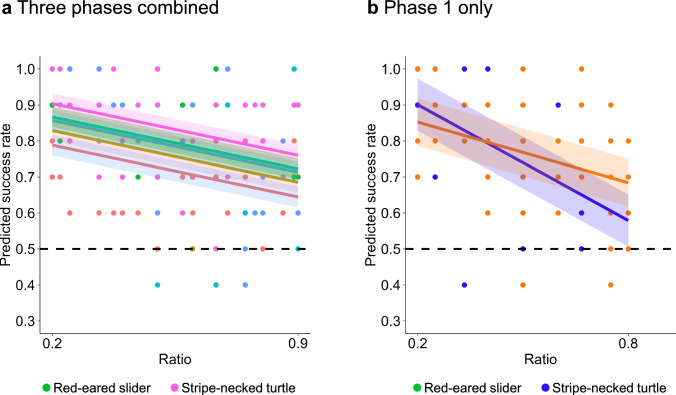
Comparison of ratio effects between red-eared slider (*Trachemys scripta elegans*, orange) and stripe-necked turtle (*Mauremys sinensis*, blue) in the mixed numerosity tests (Experiment 2). **a** The success rates between the two turtle species did not exhibit notable differences when all three phases were combined (Table S3). The performance of both species adhered to Weber’s law, where an increased ratio led to decreased performance (P < 0.001). **b** When analyzing the data separately by phase, the stripe-necked turtles showed a more pronounced decrease in performance during high-ratio trials compared to the sliders during the first phase (P = 0.0476)

In the mixed numerosity tests, the success rates between the two turtle species did not exhibit notable differences when all three phases were combined (Table S3). The performance of both species adhered to Weber’s law, where an increased ratio led to decreased performance (P < 0.001; Fig. 3a). However, when analyzing the data separately by phase, the stripe-necked turtles showed a more pronounced decrease in performance during high-ratio (more challenging) trials compared to the sliders during the first phase (t = 2.006, P = 0.0476; Fig. 3b and Table S3). Although both species displayed a negative ratio effect, the decline in the performance of the stripe-necked turtles was more marked when the ratio increased in the initial phase. These findings align with the results from the fixed numerosity test.

## Discussion

### Performance of the red-eared sliders

Our findings reaffirm the remarkable ability of freshwater turtles to discern numerical differences as close as 9 vs 10 (ratio = 0.9; Table 1). Present data suggests that red-eared sliders’ numerical discrimination surpasses that of guppies (4 vs 5 as per Bisazza et al. 2014), lizards (2 vs 3 in Miletto Petrazzini et al. 2018; 3 vs 4 in Szabo et al. 2021b), tortoises (3 vs 4 in Gazzola et al. 2018), and pigeons (6 vs 7 in Emmerton and Delius 1993). Their capability is akin to the stripe-necked turtle’s prowess (9 vs 10 as observed in Lin et al. 2021). The training techniques we adopted seemed to effectively reflected the turtles’ quantitative capabilities, illustrating their true potential.

Our study included a variety of “transfer” and “conflict” tests, as described by Howard et al. (2018). These tests would be challenging to navigate without a firm grasp on the “greater than” concept. “Transfer tests” pushed turtles with novel numerical pairings, exemplified by the progression from 1 vs 3 to 2 vs 4. “Conflict tests” required turtles to override a previous preference (such as choosing 4 as the larger in a 3 vs 4 matchup) in favor of a new one for accuracy (opting for 5 over 4 in a 4 vs 5 situation). The red-eared sliders exhibited a consistent capacity to adapt and refine their accuracy throughout the experiment. Remarkably, by the onset of Experiment 2, they adeptly tackled recurring daily challenges posed by both transfer and conflict tests.

The results of mixed numerosity tests supported the prediction of Weber’s law. As the presented ratios broadened (from 0.2 to 0.9), there was a noticeable decline in the turtles’ performance over distinct numerical pairings, spanning a mix of smaller and larger numbers. This consistent ratio-based pattern in numerical discernment was uniform among the subjects and remained unaffected by continued learning. Our research bolsters the accumulating evidence suggesting Weber’s law’s applicability across diverse vertebrate classes, from fish (as noted by Gómez-Laplaza and Gerlai 2011) and amphibians (Krusche et al. 2010) to reptiles (Gazzola et al. 2018), birds (Ditz and Nieder 2016), and primates (Jordan and Brannon 2006).

### Comparison between the two turtles

The quantity discrimination performance did not differ for both turtle species in both fixed and mixed numerosity tests. In the fixed numerosity tests for the most challenging pairs, the median success rate for the 6 vs 7 pairing was 0.640 for the stripe-necked turtle (P < 0.05, Table 1 in Lin et al. 2021) and 0.677 for the red-eared sliders (P < 0.01, Table 1). In the mixed numerosity tests, the performance for the 8 vs 9 and 9 vs 10 pairings was 0.800 for both species (P < 0.01, Table 1).

However, the red-eared sliders demonstrated a more moderate decline in performance during high ratio tests compared to the stripe-necked turtles. In the fixed numerosity test, the performance of an individual was influenced by conflicting factors: the increasing difficulty of tests diminished the performance, but the ongoing learning and practice improved it. This dynamic resulted in a progressive reduction in performance, as observed in the stripe-necked turtle in Lin et al. (2021). Still, the red-eared slider seemed less impacted by these growing challenges (Fig. 2). It suggests that an enhanced learning capability in the red-eared slider might counterbalance the heightened difficulty.

In the mixed numerosity test, the sliders faced varied difficulties concurrently. In this experiment, each turtle encountered at least 10 distinct numerosity pairs in a short time span (usually within 12–15 min). Given the pseudo-randomized sequence of the tests, the turtles were exposed to either transfer or conflict tests in each session (Table S1). Particularly in the early stages of this experiment, the red-eared slider exhibited a notably moderate negative trend compared to the stripe-necked turtle (Fig. 3b).

Yet, this disparity diminished during the second and third phases of the mixed numerosity tests. As the experiment progressed, the stripe-necked turtles gradually matched the performance of the sliders. Such a performance “catch-up” dynamic has been documented in studies examining differences between native and invasive species, as seen with the invasive green crab (Roudez et al. 2008) and the invasive gray squirrel (Chow et al. 2018). For instance, despite similar 10-day memory retention between the invasive green crab and the native blue crab, the green crab learned faster in the first 5 days (Roudez et al. 2008). Similarly, the invasive gray squirrel had superior problem-solving skills during their initial encounters compared to the native red squirrel, even if their overall capabilities were on par (Chow et al. 2018).

### Cognition and invasion success

Our study emphasizes that the red-eared sliders are adept at quantity discrimination, suggesting a new angle on understanding the mechanisms of invasion success. Historically, research on this topic has primarily focused on life history traits (e.g., Allen et al. 2017). Yet, there’s growing evidence that cognitive abilities play a significant role in an animal’s fitness, particularly in terms of survival and reproduction (Cole et al. 2012; Huebner et al. 2018; Madden et al. 2018). These abilities have shown to be crucial during the establishment phase of species introductions, influencing their invasion success (Blackburn et al. 2011; Szabo et al. 2020). Importantly, some globally recognized invasive species, including common mynas (Griffin and Diquelou 2015) and grey squirrels (Chow et al. 2018), have been found to exhibit superior cognitive skills compared to native counterparts. Our findings on the red-eared slider add to the growing discourse on the potential cognitive advantages of invasive species (Szabo et al. 2020).

For a species to become successfully invasive, behavioral flexibility is paramount. This capacity, which allows individuals to adapt behavior in response to environmental changes or to address novel challenges, plays a crucial role in their adaptability to unfamiliar settings (Shettleworth 1998; Reader and Laland 2002; Wright et al. 2010; Sol et al. 2002). Szabo et al. (2020) further emphasized that animals not only display flexibility in behaviors but also in their cognitive judgments, making optimal decisions under varying conditions. Our experiments showcase scenarios wherein turtles demonstrated this flexibility during mixed tests with inconsistent testing pairs. This research underscores the significance of cognitive studies in understanding the adaptability of invasive species and potential links between invasion success and cognitive flexibility.

### Future directions

We recognize the necessity of refining our methods to clearly differentiate between the turtles’ judgments based on quantity and those influenced by the continuous attributes of our stimuli, such as total surface area, convex hull, and density (Agrillo et al. 2011; Gebuis and Reynvoet 2012; Leibovich-Raveh et al. 2021). A limitation in our design is the direct correlation between the quantity and both the total surface area and convex hull of our stimuli, given that the cubes were of uniform size with consistent spacing. Consequently, turtles might perceive the collective size of the cubes, potentially viewing them as a singular entity (Henik et al. 2017). To ensure more robust results in future research, we recommend: (1) adjusting the size of individual objects to reduce reliance on non-numerical cues; (2) altering the object’s shape to validate the understanding of the “greater than” concept; and (3) recording and comparing the time the turtles take to make their choice. Incorporating these adjustments in experimental designs would provide clearer insights into the turtles’ decision-making processes.

In summary, our research sheds light on the numerosity discernment prowess of the red-eared slider, aligning it with the stripe-necked turtle from our prior research. The experimental design we employed underscores its replicability and broad applicability across diverse freshwater testudines under a uniform trial setup. We further elucidated that the red-eared slider, when met with high-ratio (intrinsically challenging) tasks, performs less well. This decrement manifests in both fixed numerosity tests (progressive complexity) and mixed numerosity tests (interspersed difficulty levels). Contrasted with the stripe-necked turtle, which exhibited marked susceptibility to this ratio effect, the red-eared slider potentially boasts superior resilience, equipping it to navigate multifaceted hurdles and acclimate to ever-shifting surroundings. Compared to the stripe-necked turtle, which was profoundly affected by the ratio effect, the red-eared slider may possess a greater ability to handle diverse challenges and adapt to changing environments.

## Supplementary Information

Below is the link to the electronic supplementary material.

Supplementary file 1 (DOCX 490 KB)

Supplementary file 2 (MP4 34888 KB)

Supplementary file 3 (MP4 17709 KB)

Supplementary file 4 (MP4 27617 KB)

Supplementary file 5 (XLSX 104 KB)

## Data Availability

All the data has been provided in the supplementary information which accompanies in this paper.

## Code availability

R scripts are provided in the supplementary information which accompanies in this paper.
