# Supplementary material for: Trained quantity discrimination in the invasive red-eared slider and a comparison with the native stripe-necked turtle
Source: Anim Cogn. 2024 Mar 26;27(1):26. doi: 10.1007/s10071-024-01850-0 (PMC10965720; doi:10.1007/s10071-024-01850-0)
Supplement: Supplementary file 1 — (DOCX 490 KB) [file 10071_2024_1850_MOESM1_ESM.docx]

**sSupporting Information**

**Trained quantity discrimination in invasive red-eared slider and a comparison with the native stripe-necked turtle**

Feng-Chun LIN [kame224339@gmail.com](mailto:kame224339@gmail.com)

1 Department of Zoology, University of Otago, Dunedin, New Zealand

2 School of Life Science, National Taiwan Normal University, Taipei, Taiwan

ORCID: <https://orcid.org/0000-0003-3762-3335>

Pei-Jen Lee SHANER [pshaner@ntnu.edu.tw](mailto:pshaner@ntnu.edu.tw)

1 School of Life Science, National Taiwan Normal University, Taipei, Taiwan

2 Department of Natural Resources and Environmental Studies, Hualien, National Dong Hwa University, Taiwan

ORCID: <https://orcid.org/0000-0001-8112-4299>

Ming-Ying HSIEH [mina.m.hsieh@gmail.com](mailto:mina.m.hsieh@gmail.com)

The Thinking Dog Vet Behaviour Team, Taipei, Taiwan

Martin J. WHITING [martin.whiting@mq.edu.au](mailto:martin.whiting@mq.edu.au)

Department of Biological Sciences, Macquarie University, Sydney, NSW, Australia

ORCID: <https://orcid.org/0000-0002-4662-0227>

Si-Min LIN (corresponding author) [lizard.dna@gmail.com](mailto:lizard.dna@gmail.com)

School of Life Science, National Taiwan Normal University, Taipei, Taiwan

ORCID: <https://orcid.org/0000-0001-7080-706X>

TEL: +886-2-77496246

Address: No. 88, Tingzhou Road Section 4, Taipei 116, Taiwan

**Table S1.** The pseudo-randomized sequence of tests applied in Experiment 2.

Phase 1, Day 1 – 5

| Tests | Day 1 | Day 2 | Day 3 | Day 4 | Day 5 |
| --- | --- | --- | --- | --- | --- |
| 1 | 2 vs 4 | 1 vs 3 | 2 vs 1 | 1 vs 4 | 4 vs 1 |
| 2 | 4 vs 1 | 4 vs 2 | 2 vs 5 | 4 vs 5 | 4 vs 2 |
| 3 | 5 vs 2 | 2 vs 5 | 4 vs 5 | 4 vs 2 | 3 vs 5 |
| 4 | 3 vs 5 | 5 vs 3 | 3 vs 2 | 3 vs 5 | 4 vs 3 |
| 5 | 3 vs 4 | 4 vs 3 | 3 vs 5 | 5 vs 2 | 2 vs 3 |
| 6 | 3 vs 1 | 1 vs 2 | 3 vs 1 | 5 vs 1 | 4 vs 5 |
| 7 | 2 vs 3 | 4 vs 5 | 4 vs 3 | 3 vs 4 | 5 vs 2 |
| 8 | 1 vs 2 | 4 vs 1 | 1 vs 5 | 3 vs 1 | 1 vs 5 |
| 9 | 5 vs 1 | 2 vs 3 | 4 vs 2 | 1 vs 2 | 1 vs 2 |
| 10 | 5 vs 4 | 5 vs 1 | 1 vs 4 | 3 vs 2 | 3 vs 1 |
| 11 | 2 vs 4 | 3 vs 2 | 1 vs 2 | 1 vs 4 | 1 vs 2 |
| 12 | 5 vs 1 | 1 vs 5 | 5 vs 3 | 5 vs 1 | 5 vs 3 |
| 13 | 4 vs 3 | 1 vs 4 | 5 vs 1 | 3 vs 2 | 2 vs 4 |
| 14 | 3 vs 5 | 5 vs 3 | 1 vs 3 | 1 vs 3 | 5 vs 1 |
| 15 | 5 vs 4 | 2 vs 4 | 4 vs 2 | 5 vs 4 | 4 vs 3 |
| 16 | 3 vs 1 | 1 vs 2 | 4 vs 3 | 2 vs 5 | 2 vs 5 |
| 17 | 1 vs 4 | 4 vs 3 | 2 vs 3 | 1 vs 2 | 3 vs 1 |
| 18 | 2 vs 3 | 5 vs 4 | 4 vs 1 | 4 vs 3 | 4 vs 5 |
| 19 | 2 vs 1 | 2 vs 5 | 4 vs 5 | 2 vs 4 | 4 vs 1 |
| 20 | 2 vs 5 | 3 vs 1 | 2 vs 5 | 5 vs 3 | 2 vs 3 |

**Table S1 (continued).**

Phase 2, Day 6 – 10

| Tests | Day 6 | Day 7 | Day 8 | Day 9 | Day 10 |
| --- | --- | --- | --- | --- | --- |
| 1 | 2 vs 10 | 9 vs 3 | 6 vs 4 | 4 vs 8 | 6 vs 2 |
| 2 | 6 vs 4 | 8 vs 4 | 2 vs 6 | 10 vs 6 | 4 vs 10 |
| 3 | 2 vs 6 | 6 vs 10 | 4 vs 8 | 4 vs 6 | 10 vs 8 |
| 4 | 8 vs 2 | 6 vs 8 | 8 vs 2 | 9 vs 6 | 10 vs 6 |
| 5 | 6 vs 9 | 9 vs 6 | 6 vs 10 | 6 vs 2 | 6 vs 9 |
| 6 | 10 vs 6 | 2 vs 6 | 10 vs 8 | 3 vs 9 | 8 vs 2 |
| 7 | 8 vs 4 | 8 vs 10 | 2 vs 10 | 8 vs 6 | 6 vs 8 |
| 8 | 6 vs 8 | 10 vs 4 | 9 vs 3 | 2 vs 10 | 10 vs 2 |
| 9 | 3 vs 9 | 4 vs 6 | 4 vs 10 | 8 vs 10 | 3 vs 9 |
| 10 | 10 vs 4 | 2 vs 10 | 6 vs 9 | 10 vs 4 | 4 vs 6 |
| 11 | 8 vs 10 | 8 vs 2 | 8 vs 6 | 2 vs 8 | 8 vs 4 |
| 12 | 9 vs 6 | 4 vs 8 | 6 vs 10 | 6 vs 2 | 4 vs 10 |
| 13 | 2 vs 8 | 9 vs 3 | 6 vs 2 | 4 vs 8 | 6 vs 4 |
| 14 | 8 vs 10 | 8 vs 2 | 2 vs 8 | 10 vs 8 | 9 vs 6 |
| 15 | 6 vs 2 | 8 vs 10 | 4 vs 10 | 6 vs 10 | 2 vs 10 |
| 16 | 6 vs 10 | 10 vs 4 | 10 vs 2 | 8 vs 2 | 8 vs 10 |
| 17 | 10 vs 2 | 10 vs 6 | 8 vs 6 | 6 vs 8 | 8 vs 4 |
| 18 | 10 vs 4 | 6 vs 8 | 4 vs 8 | 4 vs 6 | 3 vs 9 |
| 19 | 6 vs 8 | 9 vs 6 | 9 vs 3 | 9 vs 6 | 6 vs 2 |
| 20 | 4 vs 6 | 2 vs 10 | 6 vs 4 | 4 vs 10 | 2 vs 8 |
| 21 | 8 vs 4 | 6 vs 2 | 6 vs 9 | 10 vs 2 | 10 vs 6 |
| 22 | 3 vs 9 | 4 vs 6 | 10 vs 8 | 9 vs 3 | 8 vs 6 |

**Table S1 (continued).**

Phase 3, Day 11 – 15

| Tests | Day 11 | Day 12 | Day 13 | Day 14 | Day 15 |
| --- | --- | --- | --- | --- | --- |
| 1 | 4 vs 7 | 8 vs 3 | 3 vs 8 | 7 vs 4 | 4 vs 9 |
| 2 | 7 vs 6 | 6 vs 7 | 6 vs 3 | 8 vs 9 | 6 vs 7 |
| 3 | 4 vs 9 | 10 vs 5 | 2 vs 9 | 9 vs 10 | 9 vs 7 |
| 4 | 9 vs 8 | 10 vs 9 | 5 vs 10 | 6 vs 3 | 2 vs 9 |
| 5 | 7 vs 9 | 2 vs 9 | 10 vs 9 | 7 vs 10 | 10 vs 7 |
| 6 | 5 vs 10 | 8 vs 9 | 9 vs 8 | 9 vs 7 | 8 vs 3 |
| 7 | 10 vs 9 | 7 vs 4 | 4 vs 7 | 5 vs 10 | 9 vs 10 |
| 8 | 2 vs 9 | 7 vs 9 | 9 vs 7 | 9 vs 2 | 6 vs 3 |
| 9 | 6 vs 3 | 9 vs 4 | 9 vs 4 | 9 vs 4 | 4 vs 7 |
| 10 | 8 vs 3 | 10 vs 7 | 7 vs 10 | 3 vs 8 | 10 vs 5 |
| 11 | 7 vs 10 | 3 vs 6 | 6 vs 7 | 7 vs 6 | 8 vs 9 |
| 12 | 10 vs 5 | 9 vs 7 | 9 vs 2 | 10 vs 5 | 7 vs 6 |
| 13 | 9 vs 2 | 9 vs 2 | 8 vs 9 | 3 vs 6 | 3 vs 6 |
| 14 | 3 vs 6 | 7 vs 10 | 8 vs 3 | 10 vs 9 | 10 vs 9 |
| 15 | 7 vs 4 | 4 vs 9 | 4 vs 9 | 2 vs 9 | 7 vs 4 |
| 16 | 8 vs 9 | 7 vs 6 | 7 vs 4 | 10 vs 7 | 5 vs 10 |
| 17 | 9 vs 7 | 9 vs 10 | 3 vs 6 | 6 vs 7 | 7 vs 9 |
| 18 | 10 vs 7 | 5 vs 10 | 10 vs 7 | 9 vs 8 | 9 vs 4 |
| 19 | 3 vs 8 | 6 vs 3 | 7 vs 6 | 7 vs 9 | 3 vs 8 |
| 20 | 9 vs 4 | 4 vs 7 | 7 vs 9 | 4 vs 7 | 9 vs 8 |
| 21 | 6 vs 7 | 3 vs 8 | 10 vs 5 | 8 vs 3 | 7 vs 10 |
| 22 | 9 vs 10 | 9 vs 8 | 9 vs 10 | 4 vs 9 | 9 vs 2 |

**Table S2.** Ratio effect of stripe-necked turtle in fixed numerosity tests. Data reanalyzed from Lin et al., 2021.

| **Ratio effect of stripe-necked turtle** | | | | | | |
| --- | --- | --- | --- | --- | --- | --- |
|  | Fixed effect | Estimate | SE | df | t | P |
|  | (intercept) | 0.84302 | 0.03366 | 23 | 25.049 | <0.001*** |
|  | Ratio | -0.20760 | 0.04980 | 23 | -4.162 | 0.00038*** |
|  | Random effect | Variance | SD |  |  |  |
|  | Subject | 0 | 0 |  |  |  |
|  | (Residual) | 0.00245 | 0.04946 |  |  |  |

**Table S3.** Cross species comparison of ratio effects in mixed numerosity tests.

| **Phase 1 – 3 combined** | | | | | | |
| --- | --- | --- | --- | --- | --- | --- |
|  | Fixed effect | Estimate | SE | df | t | P |
|  | (Intercept) | 0.95880 | 0.03941 | 16.08561 | 24.33 | <0.001*** |
|  | Species TS (vs MS) | -0.04958 | 0.04430 | 76.31139 | -1.119 | 0.267 |
|  | Ratio | -0.33265 | 0.05058 | 338.54444 | -6.576 | <0.001*** |
|  | Species TS: Ratio  (vs MS: Ratio) | 0.09514 | 0.06755 | 336.98738 | 1.408 | 0.16 |
|  | Random effect | Variance | SD |  |  |  |
|  | Subject | 0.00119 | 0.03444 |  |  |  |
|  | Phase | 0.00139 | 0.03730 |  |  |  |
|  | (Residual) | 0.01775 | 0.13321 |  |  |  |
| **Phase 1** | | | | | | |
|  | Fixed effect | Estimate | SE | df | t | p |
|  | (Intercept) | 1.00847 | 0.05252 | 91.55419 | 19.201 | <0.001*** |
|  | Species TS (vs MS) | -0.09962 | 0.07111 | 91.55419 | -1.401 | 0.1646 |
|  | Ratio | -0.53693 | 0.09421 | 97 | -5.699 | <0.001*** |
|  | Species TS: Ratio  (vs MS: Ratio) | 0.25591 | 0.12756 | 97 | 2.006 | 0.0476* |
|  | Random effect | Variance | SD |  |  |  |
|  | Subject | 0.00101 | 0.03177 |  |  |  |
|  | (Residual) | 0.01689 | 0.12995 |  |  |  |
| **Phase 2** | | | | | | |
|  | Fixed effect | Estimate | SE | df | t | P |
|  | (Intercept) | 0.94185 | 0.05318 | 89.80615 | 17.709 | <0.001*** |
|  | Species TS (vs MS) | 0.00598 | 0.07201 | 89.80615 | 0.083 | 0.93405 |
|  | Ratio | -0.31643 | 0.09373 | 108 | -3.376 | 0.00102** |
|  | Species TS: Ratio  (vs MS: Ratio) | -0.00650 | 0.12690 | 108 | -0.051 | 0.95927 |
|  | Random effect | Variance | SD |  |  |  |
|  | Subject | 0.00142 | 0.03769 |  |  |  |
|  | (Residual) | 0.01916 | 0.13840 |  |  |  |
| **Phase 3** | | | | | | |
|  | Fixed effect | Estimate | SE | df | t | P |
|  | (Intercept) | 0.96632 | 0.05320 | 103.75873 | 18.165 | <0.001*** |
|  | Species TS (vs MS) | -0.10925 | 0.07203 | 103.75873 | -1.517 | 0.13236 |
|  | Ratio | -0.24783 | 0.07903 | 108 | -3.136 | 0.00221** |
|  | Species TS: Ratio  (vs MS: Ratio) | 0.13979 | 0.10701 | 108 | 1.306 | 0.19421 |
|  | Random effect | Variance | SD |  |  |  |
|  | Subject | 0.00097 | 0.03107 |  |  |  |
|  | (Residual) | 0.01617 | 0.12717 |  |  |  |

*: P < 0.05; **: P < 0.01; ***: P < 0.001. Abbreviations: TS: the red-eared slider (*Trachemys scripta elegans*); MS: the stripe-necked turtle (*Mauremys sinensis*).

**Figure S1.** Comparison of ratio effects in the mixed-numerosity tests (Experiment 2) between the red-eared slider (*Trachemys scripta elegans*, represented in orange) and the stripe-necked turtle (*Mauremys sinensis*, represented in blue), illustrated through the sequence of the three phases.
